# Survival benefits of interferon-based therapy in patients with recurrent hepatitis C after orthotopic liver transplantation

**DOI:** 10.1590/1414-431X20165540

**Published:** 2017-01-09

**Authors:** L.P. Zanaga, A.G. Vigani, R.N. Angerami, A. Giorgetti, C.A.F. Escanhoela, E.C. Ataíde, I.F.S.F. Boin, R.S.B. Stucchi

**Affiliations:** 1 Disciplina de Infectologia, Departamento de Clínica Médica, Faculdade de Ciências Médicas, Universidade Estadual de Campinas, Campinas, SP, Brasil; 2 Departamento de Anatomia Patológica, Faculdade de Ciências Médicas, Universidade Estadual de Campinas, Campinas, SP, Brasil; 3 Unidade de Transplante de Fígado, Departamento de Cirurgia, Faculdade de Ciências Médicas, Universidade Estadual de Campinas, Campinas, SP, Brasil

**Keywords:** Hepatitis C, Liver transplantation, Sustained virological response, Recurrent hepatitis C, Transplantation outcomes

## Abstract

Recurrent hepatitis C after orthotopic liver transplantation (OLT) is universal and can lead to graft failure and, consequently, reduced survival. Hepatitis C treatment can be used to prevent these detrimental outcomes. The aim of this study was to describe rates of hepatitis C recurrence and sustained virological response (SVR) to interferon-based treatment after OLT and its relationship to survival and progression of liver disease through retrospective analysis of medical records of 127 patients who underwent OLT due to cirrhosis or hepatocellular carcinoma secondary to chronic hepatitis C between January 2002 and December 2013. Fifty-six patients were diagnosed with recurrent disease, 42 started interferon-based therapy and 37 completed treatment. Demographic, treatment- and outcome-related variables were compared between SVR and non-responders (non-SVR). There was an overall 54.1% SVR rate with interferon-based therapies. SVR was associated with longer follow-up after treatment (median 66.5 *vs* 37 months for non-SVR, P=0.03) and after OLT (median 105 *vs* 72 months, P=0.074), and lower rates of disease progression (15 *vs* 64.7%, P=0.0028) and death (5 *vs* 35.3%, P=0.033). Regardless of the result of therapy (SVR or non-SVR), there was a significant difference between treated and untreated patients regarding the occurrence of death (P<0.001) and months of survival (P<0.001). Even with suboptimal interferon-based therapies (compared to the new direct-acting antivirals) there is a 54.1% SVR rate to treatment. SVR is associated with improved survival and reduced risks of clinical decompensation, loss of the liver graft and death.

## Introduction

Chronic hepatitis C virus (HCV) infection leading to decompensated liver cirrhosis or hepatocellular carcinoma is the main cause of orthotopic liver transplantation (OLT) worldwide. It is expected that the number of patients with HCV infection referred for OLT will continue to increase in the next years, in spite of advances in antiviral therapy (1).

Nonetheless, if HCV viremia is present during the transplantation procedure, the result is universal reinfection of liver allografts, happening as early as the reperfusion phase of the surgical procedure, with viral replication within hours after OLT (2,3). Recurrent liver disease due to HCV usually develops after 3 months and is present in up to 70–90% of patients 1 year after OLT. Furthermore, the progression of recurrent disease is faster than in the immunocompetent population (4–7). Recurrent liver disease associated with HCV infection leads to consequent graft loss in about one third of patients within 5 years of OLT (6,8) and graft failure due to recurrent HCV is the main cause of patient death and retransplantation by the 5th postoperative year (9). Therefore, survival of patients with chronic HCV infection is significantly reduced when compared to other causes of OLT (4–8,10).

The virological efficacy of HCV therapeutic options has improved drastically over recent years, from 30% success rate with interferon-based therapies to around 90% with interferon-free direct acting antiviral agents (DAAs) (11). However, regardless of the medication used, the objectives of HCV treatment have not changed: to prevent progression to cirrhosis and loss of the graft (12–20). In HCV-infected patients, the achievement of sustained virological response (SVR) after treatment reduces the risk of progression to clinical decompensation or development of hepatocellular carcinoma in cirrhotic patients and can even result in histological improvement in those with less advanced fibrosis. Some studies have evaluated this benefit in post-OLT patients as well as the impact on survival, but studies of long-term outcomes are lacking (10,12–16,21–25).

The aim of this study is to describe rates of hepatitis C recurrence and SVR to interferon-based treatment after OLT and its relationship to survival and progression of liver disease in a group of patients transplanted due to end-stage chronic HCV infection in a single center in Brazil.

## Material and Methods

### Patient selection

This study included adult patients (age ≥18 years) who underwent OLT due to cirrhosis or hepatocellular carcinoma secondary to chronic HCV infection from January 2002 to December 2013 at the Hospital de Clínicas of the Universidade Estadual de Campinas, Brazil, with positive anti-HCV serology and HCV-RNA. A retrospective analysis of the patients’ medical records was performed. The follow-up period ended at the time of the patient's death or at the end of the observation period (July 2014) and was the basis for the evaluation of survival. The exclusion criteria were coinfection with hepatitis B virus (detectable hepatitis B surface antigen), negative HCV-RNA before OLT, use of alcohol or illicit drugs after OLT, follow up at another transplant unit, incomplete medical records and survival after OLT shorter than 1 month (to rule out cases of early mortality related to the surgical procedure).

Recurrent hepatitis C after liver transplantation was defined as the presence of detectable serum HCV-RNA assessed by polymerase chain reaction (PCR; qualitative or quantitative) and compatible histology (for differential diagnosis with other complications, such as rejection, biliary disease or vascular complications).

### Histological examination

Liver biopsies were not routinely scheduled, but performed after the detection of elevated liver transaminases during follow-up. Biopsies were considered compatible with recurrent hepatitis C based on findings of portal or lobular infiltration by mononuclear cells with piecemeal necrosis and were graded according to the Metavir score. If histology presented mixed portal infiltrate, venous endothelitis and bile duct injury, acute rejection was diagnosed. Chronic rejection was considered when there was bile duct atrophy, paucity or foam cell obliterative arteriopathy. If the biopsy was compatible with rejection and diagnosed during or immediately after stopping treatment, the case was analyzed by the assistant physician to define if its occurrence was associated to HCV therapy.

### Antiviral treatment regimen

Antiviral treatment regimen consisted of ribavirin (RBV, 15 mg/kg daily) associated with pegylated interferon (PegIFN, α2a 180 µg or α2b 1.5 µg/kg weekly) or conventional interferon alpha (IFN, 3 million IU three times a week). Local protocols established that patients should be treated for 12 months after achieving HCV-RNA negativity. For patients who have been retreated, the information collected was that of the most recent regimen. SVR was defined as negative HCV-RNA 24 weeks after the completion of therapy.

Adjunctive medication could be used for the management of side effects, such as erythropoietin (doses up to 40.000 UI weekly) if hemoglobin ≤10 g/dL, and filgrastim (300 µg weekly) if neutrophils ≤750/mm^3^. The dosage of IFN, PegIFN, and RBV could also be decreased. Absolute contraindications for HCV treatment were the presence of rejection at the beginning of treatment, decompensated cirrhosis (Child-Pugh B or C), severely low platelets (<30,000/mm^3^) and psychiatric comorbidities.

### Immunosuppression

Immunosuppression was managed according to the internal guidelines, consisting of corticosteroids (generally withdrawn within 6 months after OLT) and a calcineurin inhibitor as the main immunosuppressive agent (cyclosporine or tacrolimus), at times associated to mycophenolate mofetil. Acute rejection episodes were managed with high doses of intravenous corticosteroids (methylprednisolone 1 g daily for 3 days). Chronic rejection was managed with steroids and alteration of the main immunosuppressive agent. When rejection was diagnosed before the start of interferon-based therapy, the antiviral treatment was postponed until rejection episodes were controlled.

### Data collection

Data regarding patient characteristics (age, gender, body mass index, comorbidities), surgical procedures, laboratory and biopsy results, use of medication (immunosuppression and HCV therapy) and clinical follow-up were collected using a standardized form.

### Endpoints

Four endpoints were analyzed: 1) HCV recurrence after OLT, 2) virological response to therapy, 3) occurrence of progression of liver disease, and 4) survival after treatment.

Progression of disease post-treatment was defined by the presence of worsening of fibrosis on graft biopsy or the development of clinical decompensation, such as hepatic encephalopathy, jaundice, ascites, spontaneous peritonitis, esophageal hemorrhage or hepatocellular carcinoma (HCC).

### Statistical analysis

Statistical analysis was performed using EpiInfo Software version 7.1.5.2 (CDC, USA). Categorical data are reported as percentages and continuous variables are reported as medians with ranges. The chi-square or Fisher's exact test was used to compare categorical data, when appropriate. The Kruskal-Wallis method was used to analyze continuous data. Overall survival was calculated by Kaplan-Meier survival curves with log-rank survival comparisons and 95% confidence intervals. Variables for which an association was suspected (P<0.2) in the univariate analysis were included in a stepwise logistic regression model. P≤0.05 was considered to be significant.

### Ethical considerations

The study was approved by the Ethics Committee of the Faculty of Medical Sciences of the Universidade Estadual de Campinas.

## Results

From January 2002 to December 2013, 193 patients underwent OLT at the Universidade Estadual de Campinas due to cirrhosis or hepatocellular carcinoma secondary to chronic HCV infection and 127 (65.8%) met the inclusion criteria for the study. One patient was excluded because of incomplete medical records, 2 patients were excluded because they were referred to another hospital for follow-up, 3 patients as a result of narcotics use after OLT, 4 due to coinfection with hepatitis B virus, 11 because of negative HCV-PCR before OLT, and 45 due to survival of less than 1 month after OLT.

### Demographics and pretreatment patient characteristics

The patients were mostly male (76.4%) and at OLT the median age was 52 years (range: 24–70 years), median body mass index of 26 (range: 18–42), median model for end-stage liver disease (MELD) (without adjustment) of 17 (range: 7–42) and 65 (51.2%) were Child-Pugh C. HCC was present in 69 cases (54.3%), with 11 incidental tumors (15.7%). Nine patients required retransplantation (7.1% of the total), 6 (66.7%) due to arterial thrombosis, and 3 (33.3%) due to chronic rejection. The patients were followed for a median period of 33 months post-transplantation (range: 1–144).

Eighty-five patients (66.9%) were submitted to liver biopsies after the detection of elevated liver tests (aspartate aminotransferase, alanine aminotransferase, billirrubin) on routine follow-up. Fifty-six patients (44.1%) were diagnosed with recurrent hepatitis C, at a median of 12.5 months after OLT (range: 1–100 months).

**Figure 1:**
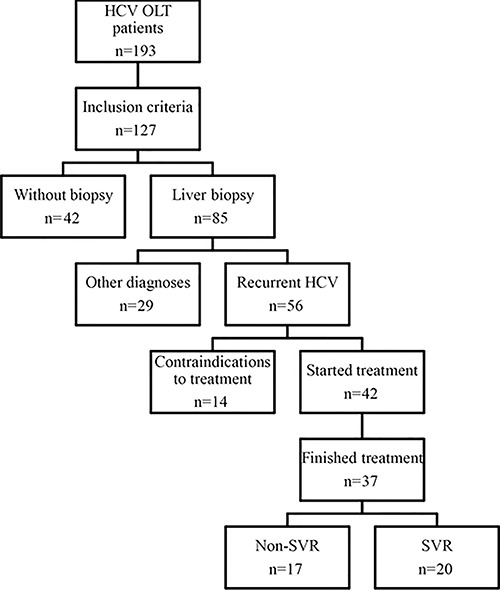
Algorithm of patient selection and treatment outcome. HCV: hepatitis C virus; OLT: orthotopic liver transplantation; SVR: sustained virological response.

Forty-two patients (33.1%) received at least one dose of treatment with either IFN or PegIFN and RBV: 37 (29.1%) completed treatment and 5 were still on treatment during data collection. Eighty-five (66.9%) patients never started treatment, 14 (11%) due to contraindications to interferon-based therapy (psychiatric disease, Child-Pugh B or C cirrhosis and uncontrolled comorbidities, such as coronary heart disease and diabetes). Seventy-one patients (55.9%) were not treated due to lack of diagnosis of recurrent HCV disease, since 29 patients’ biopsies had diagnoses other than recurrent HCV, and 42 were not submitted to biopsy, because of absence of alteration of liver transaminases or lack of clinical conditions for biopsy. The complete patient selection algorithm is shown in Figure 1. The patients’ characteristics, stratified into treated and untreated, are described in Table 1.

**Table:**
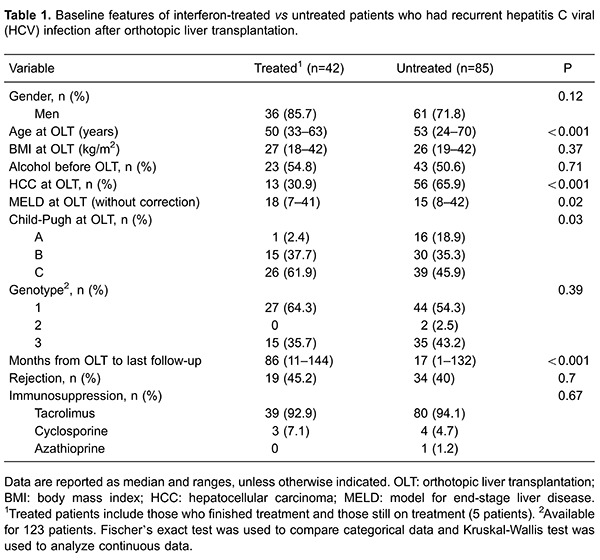

**Figure 2:**
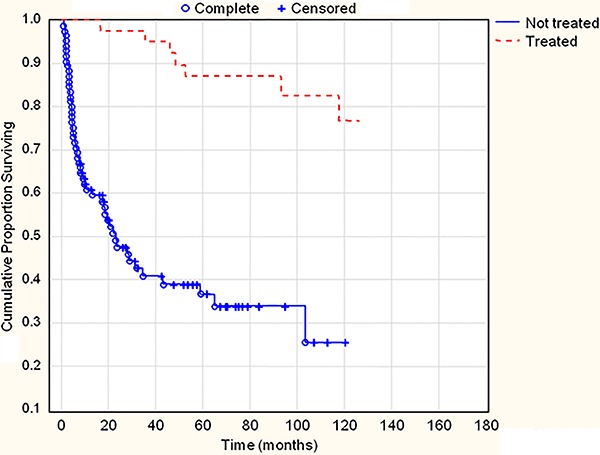
Cumulative survival (Kaplan-Meier) of interferon-treated versus untreated patients who had recurrent hepatitis C viral (HCV) infection after orthotopic liver transplantation. Survival was significantly better in those who received recurrent HCV treatment (P<0.001).

In the univariate analysis, factors associated with HCV treatment were younger age at OLT, absence of HCC before OLT, higher MELD score and Child-Pugh C (Table 1). In multivariate analysis, male gender [odds ratio (OR)= 0.29; 95% confidence interval (CI): 0.09–0.92], younger age at OLT (OR=0.94; 95%CI=0.88-0.99) and absence of HCC before OLT (OR=0.27; 95%CI=0.11–0.68) were independently and significantly associated with HCV treatment. Regardless of treatment response, death outcome was significantly more frequent among untreated (58.8%, 50 of 85 patients) than treated patients (16.7%, 7 of 42), P<0.001. There was a noteworthy difference in survival between treated and untreated patients (P<0.001, Figure 2).

### Treatment characteristics and virological response

Thirty-seven patients (66.1% of those with HCV recurrence) completed treatment. Therapy was initiated at median 18 months after OLT (range: 4–49) and the median duration was 68 weeks (range: 2–172). Sixteen patients had been treated before OLT, without achieving SVR.

Five (13.5%) patients were treated with IFN and RBV and 32 (86.5%) with PegIFN and RBV. The overall SVR rate was 54.1% (20 of 37 patients treated) and 50% of those who reached SVR had already been treated unsuccessfully before OLT. The SVR rate of patients treated with PegIFN and RBV was 46.9% (15 of 32), and was higher for genotype 3 infection (46.1%, 6 of 13 patients, versus 37.5%, 9 of 24 patients with HCV genotype 1). The characteristics of the treated patients are described in Table 2.

**Table:**
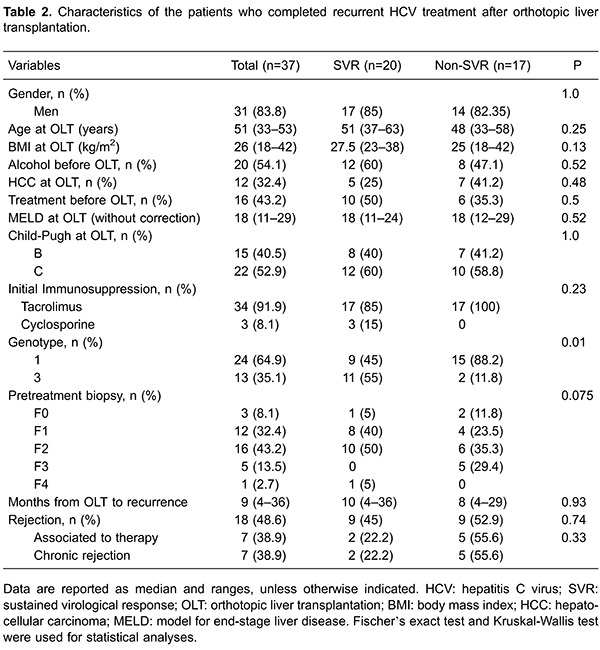

Eight patients (21.6%) were retreated. Among the patients treated with IFN and RBV, 3 were retreated with PegIFN due to previous non-response, but only 1 achieved SVR. On the other hand, 5 patients of the PegIFN group were retreated, with change in the type of medication (PegIFN α 2a or 2b), with 2 (40%) additional SVR cases. The treatment characteristics are described in Table 3.

**Table:**
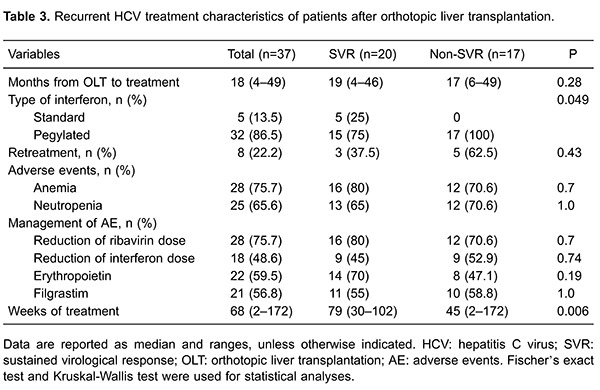

Univariate analysis revealed genotype 3, type of interferon and longer treatment duration as being significantly associated with SVR (Tables 2 and 3). In multivariate analysis, no variable was significantly associated with SVR.

### Clinical outcomes after treatment - chronic liver disease progression and survival

Among the 37 patients who completed treatment, the median duration of follow-up after treatment was 51 months (range: 1–111). Three (15%) patients with SVR had signs of progression of liver disease (one with jaundice and ascites, one with fibrosis evolution on biopsy and another with worsening of fibrosis and encephalopathy). On the other hand, among 17 non-SVR patients, 11 (64.7%) had disease progression (P=0.002; Table 4). Nine non-SVR patients had worsening of fibrosis on biopsy specimens. Three of these patients have been retreated due to previous treatment failure and had progression of fibrosis when comparing biopsies before and after the first treatment. There were no cases of esophageal variceal bleeding or HCC post OLT among the treated patients. In multivariate analysis, prevention of liver disease progression after treatment (OR=0.09; 95%CI=0.014–0.66) was independently and significantly associated with SVR.

**Table:**
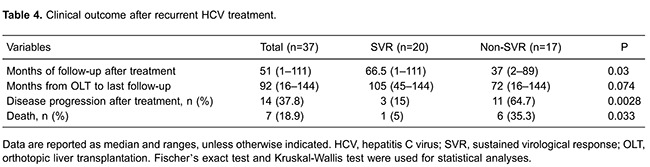

Overall, 7 patients died, 1 (5%) in the SVR group and 6 (35.3%) non-SVR, P=0.03. The only death among SVR patients was related to metastatic colonic adenocarcinoma and among non-SVR patients 4 died due to sepsis, 1 due to hepatic insufficiency, and 1 due to multiorgan failure. Median post-transplant survival was 105 months (range: 45–144) for SVR patients and 72 months (range: 16–144) for non-SVR, P=0.003 (Table 4).

**Figure 3:**
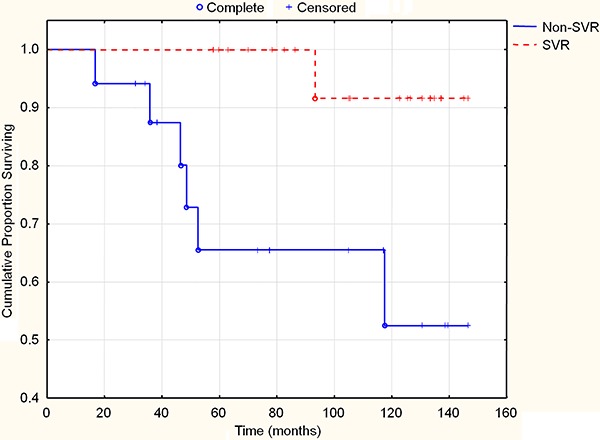
Cumulative survival (Kaplan-Meier) of patients who had recurrent hepatitis C viral (HCV) infection after orthotopic liver transplantation with sustained virological response (SVR) versus non-SVR. Survival was significantly better in those who achieve SVR after recurrent HCV therapy (P<0.001).

The Kaplan-Meier survival analysis demonstrated that patients who achieved SVR had significantly longer survival than non-SVR (P<0.001, Figure 3).

## Discussion

Recurrent hepatitis C following OLT is a challenge to physicians worldwide and is a significant threat to the survival of both the patient and his or her graft, since OLT recipients with recurrent hepatitis C have faster disease progression when compared to non-immunosuppressed individuals (4–7). However, antivirals have been used in an attempt to modify the course of HCV recurrent disease. Our study showed a significant rate of recurrent hepatitis C after OLT and the majority of patients had mild to moderate fibrosis (F1–F2) severity on liver biopsy. The factors associated with antiviral treatment were younger age at OLT, male gender and absence of HCC at OLT. Treated patients who did not achieve SVR had longer survival, and those who reached SVR had longer survival and lower rates of clinical decompensation, loss of the graft and death.

Even though there is universal recurrence of HCV after OLT, HCV was only diagnosed in 44.1% of the patients included in the study and in 65.9% if considering those who were submitted to liver biopsies after the elevation of transaminases. The use of protocol liver biopsies may result in higher rates of recurrence, as in the study by Shuhart et al. (4), who described a 66% rate. The population studied had recurrent disease diagnosed 9 months after OLT (range: 4–36), in contrast to previous studies which detected delayed-onset recurrence, ranging from 13.4 to 34 months post-OLT (4,8).

In the present study, only 21.8% of patients transplanted due to HCV cirrhosis started interferon-based therapies, which is lower than treatment rates described in previous studies, ranging from 38.6 to 68% (7,12,14,21,22,25). This difference could be explained by the lack of protocol biopsies and the presence of contraindications to treatment. The rates of contraindications are about 17.3% in the general population (26) and reached 25% of patients with recurrent HCV in our study.

Antiviral therapy with PegIFN and RBV for 48 weeks results in a SVR rate of around 30.2% (23 [Bibr B24]). The present study found a significantly higher overall SVR rate of 54.1% and this difference could be justified by the prolonged duration of treatment (median of 68 weeks) when compared to other studies in which patients were treated for 48 weeks on average (10,12,14,16,18–25,27,28). Besides, in the population studied there was a considerable difference in length of treatment among SVR and non-SVR patients.

Even though over the last decades many patients have been treated and reached SVR with interferon-based therapies, nowadays studies focus on DAAs (protease, NS5A and polymerase inhibitors) for the treatment of HCV, due to higher SVR rates, and fewer contraindications and side-effects. Data from clinical trials and real-life settings will provide information to assess the impact of therapy in the DAA era and the role of ribavirin nowadays (11). Especially for OLT recipients, these new drugs bring renewed hope, since the use of interferon and ribavirin in the post-transplantation population is associated with lower SVR rates and high rates of adverse events, such as anemia and neutropenia. This population requires modifications on the dosage of interferon or ribavirin, adjunctive therapies, like filgrastim or erythropoietin, or even treatment interruption. Patients treated were evaluated frequently (weekly if necessary), to assess for adverse events of treatment and prompt management, allowing for treatment continuation. The rates of medication dose reduction found in our population are compatible with previous studies. Another major concern regarding OLT recipients under interferon therapy is the occurrence of rejection due to immune-mediated graft dysfunction. In the population studied, 38.9% of cases of rejection were related to HCV interferon-based therapy, which was higher than rates of 0 to 25% previously described in other studies (23). Moreover, two non-SVR patients developed chronic rejection related to HCV therapy, leading to graft failure and consequent retransplantation. The ideal therapy for recurrent HCV should have high efficacy, good tolerability, lack of interaction with immunosuppressants and should not induce graft rejection.

Previous studies have shown benefits in treating this special patient population, since the achievement of SVR can lead to histological improvement (16,18–21,23,25). Therefore, the achievement of SVR is expected to reduce the occurrence of progression to chronic liver disease, manifested by progression of fibrosis on subsequent liver biopsies, diagnosis of hepatocellular carcinoma or other complications associated to cirrhosis, such as ascites, spontaneous bacterial peritonitis, esophageal variceal bleeding, hepatic encephalopathy, jaundice and loss of the graft. The present study encountered a significant difference among SVR patients and non-SVR regarding liver disease progression.

Furthermore, patient survival was overall significantly better in patients who achieved SVR, which is compatible with results from other cohort studies (10,12–16,21–25). The only death among the patients who achieved SVR was unrelated to transplantation complications or recurrent hepatitis C. It must be noted that, regardless of the result of therapy (SVR or non-SVR), there was a significant difference between treated and untreated patients regarding the occurrence of death and length of survival.

Limitations of this study include its small sample size, the retrospective design and changes in clinical management protocols (regarding immunosuppression and hepatitis treatment), which could introduce confounding factors. Another concern is the fact that liver biopsies were not performed by protocol, potentially reducing the diagnoses of recurrent hepatitis C and, consequently, the indication for treatment. A selection bias could also be involved, since interferon-based therapies have contraindications that could exclude the sickest patients from treatment. Furthermore, during the study period, access to HCV-RNA assays was limited and it was not possible to perform viral kinetics analysis. Since treatment protocol established that therapy should last for 12 months after a negative PCR was obtained, difficulty of access to the exam could have led to the prolonged treatment periods observed.

The main conclusion of this study is that the achievement of SVR is associated with improved survival and reduced risks of clinical decompensation, loss of the liver graft and death.

## References

[1] Razavi H, Waked I, Sarrazin C, Myers RP, Idilman R, Calinas F (2014). The present and future disease burden of hepatitis C virus (HCV)
infection with today's treatment paradigm. J Viral Hepat.

[2] Garcia-Retortillo M, Forns X, Feliu A, Moitinho E, Costa J, Navasa M (2002). Hepatitis C virus kinetics during and immediately after liver
transplantation. Hepatology.

[3] Gane EJ, Portmann BC, Naoumov NV, Smith HM, Underhill JA, Donaldson PT (1996). Long-term outcome of hepatitis C infection after liver
transplantation. N Engl J Med.

[4] Shuhart MC, Bronner MP, Gretch DR, Thomassen LV, Wartelle CF, Tateyama H (1997). Histological and clinical outcome after liver transplantation for
hepatitis C. Hepatology.

[B05] Vinaixa C, Rubin A, Aguilera V, Berenguer M (2013). Recurrence of hepatitis C after liver transplantation. Ann Gastroenterol.

[6] Gallegos-Orozco JF, Yosephy A, Noble B, Aqel BA, Byrne TJ, Carey EJ (2009). Natural history of post-liver transplantation hepatitis C: A review of
factors that may influence its course. Liver Transpl.

[7] Firpi RJ, Clark V, Soldevila-Pico C, Morelli G, Cabrera R, Levy C (2009). The natural history of hepatitis C cirrhosis after liver
transplantation. Liver Transpl.

[8] Ghobrial RM, Steadman R, Gornbein J, Lassman C, Holt CD, Chen P (2001). A 10-year experience of liver transplantation for hepatitis C:
analysis of factors determining outcome in over 500 patients. Ann Surg.

[9] Charlton M (2003). Natural history of hepatitis C and outcomes following liver
transplantation. Clin Liver Dis.

[10] Wawrzynowicz-Syczewska M, Zeair S, Andruszkiewicz A, Bartoszewicz K, Slawinski J, Laurans L (2014). Impact of antiviral treatment on survival in HCV-positive liver
recipients. Ann Transplant.

[11] Berenguer M (2015). Management of HCV in the liver transplant setting. Clin Res Hepatol Gastroenterol.

[12] Bizollon T, Pradat P, Mabrut JY, Chevallier M, Adham M, Radenne S (2005). Benefit of sustained virological response to combination therapy on
graft survival of liver transplanted patients with recurrent chronic hepatitis
C. Am J Transplant.

[B13] Kornberg A, Kupper B, Tannapfel A, Thrum K, Barthel E, Habrecht O (2008). Sustained clearance of serum hepatitis C virus-RNA independently
predicts long-term survival in liver transplant patients with recurrent hepatitis
C. Transplantation.

[14] Picciotto FP, Tritto G, Lanza AG, Addario L, De Luca M, Di Costanzo GG (2007). Sustained virological response to antiviral therapy reduces mortality
in HCV reinfection after liver transplantation. J Hepatol.

[B15] Faisal N, Bilodeau M, Aljudaibi B, Hirsch G, Yoshida EM, Hussaini T (2016). Sofosbuvir-antiviral therapy is highly effective in recurrent
hepatitis C in liver transplant recipients: Canadian multicenter "Real-Life"
experience. Transplantation.

[16] Berenguer M, Schuppan D (2013). Progression of liver fibrosis in post-transplant hepatitis C:
mechanisms, assessment and treatment. J Hepatol.

[B17] Berenguer M, Palau A, Aguilera V, Rayon JM, Juan FS, Prieto M (2008). Clinical benefits of antiviral therapy in patients with recurrent
hepatitis C following liver transplantation. Am J Transplant.

[18] Berenguer M, Aguilera V, Rubin A, Ortiz C, Jimenez M, Prieto M (2012). Comparison of two non-contemporaneous HCV-liver transplant cohorts:
strategies to improve the efficacy of antiviral therapy. J Hepatol.

[B19] Berenguer M, Palau A, Fernandez A, Benlloch S, Aguilera V, Prieto M (2006). Efficacy, predictors of response, and potential risks associated with
antiviral therapy in liver transplant recipients with recurrent hepatitis
C. Liver Transpl.

[20] Berenguer M, Roche B, Aguilera V, Duclos-Vallee JC, Navarro L, Rubin A (2013). Efficacy of the retreatment of hepatitis C virus infections after
liver transplantation: role of an aggressive approach. Liver Transpl.

[21] Kawaoka T, Takahashi S, Kawakami Y, Tsuge M, Hiramatsu A, Imamura M (2015). Sustained virological response to antiviral therapy improves survival
rate in patients with recurrent hepatitis C virus infection after liver
transplantation. Hepatol Res.

[22] Tanaka T, Selzner N, Therapondos G, Renner EL, Lilly LB (2013). Virological response for recurrent hepatitis C improves long-term
survival in liver transplant recipients. Transpl Int.

[23] Berenguer M (2008). Systematic review of the treatment of established recurrent hepatitis
C with pegylated interferon in combination with ribavirin. J Hepatol.

[B24] Gurusamy KS, Tsochatzis E, Toon CD, Xirouchakis E, Burroughs AK, Davidson BR (2013). Antiviral interventions for liver transplant patients with recurrent
graft infection due to hepatitis C virus. Cochrane Database Syst Rev.

[25] Selzner N, Renner EL, Selzner M, Adeyi O, Kashfi A, Therapondos G (2009). Antiviral treatment of recurrent hepatitis C after liver
transplantation: predictors of response and long-term outcome. Transplantation.

[26] Talal AH, LaFleur J, Hoop R, Pandya P, Martin P, Jacobson I (2013). Absolute and relative contraindications to pegylated-interferon or
ribavirin in the US general patient population with chronic hepatitis C: results
from a US database of over 45 000 HCV-infected, evaluated patients. Aliment Pharmacol Ther.

[27] Ponziani FR, Milani A, Gasbarrini A, Zaccaria R, Vigano R, Iemmolo RM (2013). Treatment of genotype-1 hepatitis C recurrence after liver transplant
improves survival in both sustained responders and relapsers. Transpl Int.

[28] Garcia-Reyne A, Lumbreras C, Fernandez I, Colina F, Abradelo M, Magan P (2013). Influence of antiviral therapy in the long-term outcome of recurrent
hepatitis C virus infection following liver transplantation. Transpl Infect Dis.

